# Synergy of hypoxia relief and heat shock protein inhibition for phototherapy enhancement

**DOI:** 10.1186/s12951-020-00749-5

**Published:** 2021-01-06

**Authors:** Gutian Zhang, Wenting Cheng, Lin Du, Chuanjun Xu, Jinlong Li

**Affiliations:** 1 Department of Urology, Drum Tower Hospital, Medical School of Nanjing University, Nanjing, 210008 China; 2 Department of Laboratory Medicine, The Second Hospital of Nanjing, Nanjing University of Chinese Medicine, Nanjing, 210003 China; 3 Department of Urology, Drum Tower Hospital, Medical School of Southeast University, Nanjing, 210008 China

## Abstract

**Background:**

Phototherapy is a promising strategy for cancer therapy by reactive oxygen species (ROS) of photodynamic therapy (PDT) and hyperthermia of photothermal therapy (PTT). However, the therapeutic efficacy was restricted by tumor hypoxia and thermal resistance of increased expression of heat shock protein (Hsp). In this study, we developed albumin nanoparticles to combine hypoxia relief and heat shock protein inhibition to overcome these limitations for phototherapy enhancement.

**Results:**

Near-infrared photosensitizer (IR780) and gambogic acid (GA, Hsp90 inhibitor) were encapsulated into albumin nanoparticles via hydrophobic interaction, which was further deposited MnO_2_ on the surface to form IGM nanoparticles. Both in vitro and in vivo studies demonstrated that IGM could catalyze overexpress of hydrogen peroxide to relive hypoxic tumor microenvironment. With near infrared irradiation, the ROS generation was significantly increase for PDT enhancement. In addition, the release of GA was promoted by irradiation to bind with Hsp90, which could reduce cell tolerance to heat for PTT enhancement. As a result, IGM could achieve better antitumor efficacy with enhanced PDT and PTT.

**Conclusion:**

This study develops a facile approach to co-deliver IR780 and GA with self-assembled albumin nanoparticles, which could relive hypoxia and suppress Hsp for clinical application of cancer phototherapy.

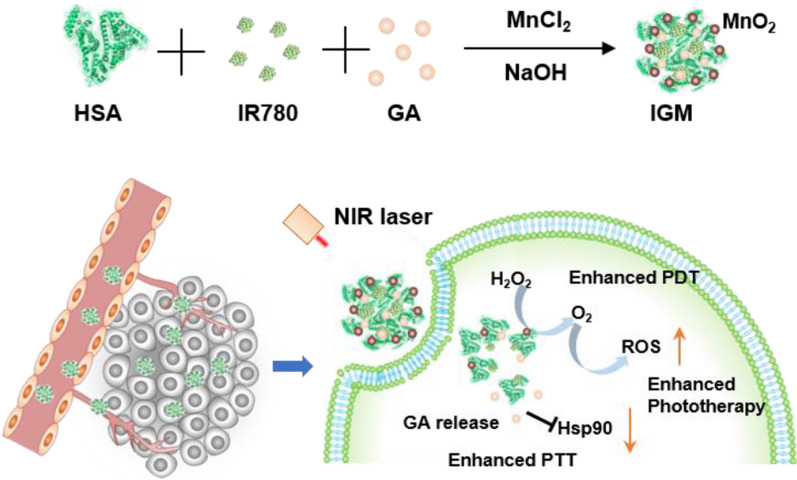

## Introduction

Phototherapy has attracted great attention for cancer treatment due to its high selectivity with laser irradiation [1, 2]. The photo-agents absorb light energy to generate heat as photothermal therapy (PTT) or reactive oxygen species (ROS) as photodynamic therapy (PDT), which could destroy cancer cells for cancer treatment [3–5]. In recent years, small organic near-infrared (NIR) photosensitizers (such as IR780, IR820 or indocyanine green) have been applied for phototherapy due to its high tissue penetration [6, 7]. They have been proved to produce heat and ROS with combination of PTT and PDT for cancer treatment [8, 9]. However, hypoxia is the main characteristic of tumor microenvironment, which limited therapeutic efficacy of the highly oxygen (O_2_)-dependent PDT [10, 11]. Oxygen delivery system or in situ generation system has been developed to reverse hypoxia, such as delivering O_2_ by perfluorocarbon with high oxygen capability or generation hydrogen peroxide (H_2_O_2_) with MnO_2_ [12–14]. Among them, various formulations of MnO_2_ have been reported to catalyze the overexpression of H_2_O_2_, which could in situ produce O_2_ and generate more ROS for PDT cancer treatment [15]. Therefore, we hypothesize that combined with MnO_2_, the NIR-induced PDT could be enhanced to effectively kill cancer cells.

The hyperthermia by PTT could effectively induce the death of tumor cells, but the high increased temperature may confer collateral damage to normal tissue [16, 17]. In order to solve this problem, scientists used mild temperature (below 45 °C) to kill cancer cells [18–20]. However, under mild hyperthermia condition, heat shock proteins (Hsp) are highly expressed to cause the heat stress tolerance of cancer cells, which lead to high chances of recurrence [21–23]. Previous studies have been reported to deliver small interfering RNA (siRNA) and ATP inhibitor (glucose oxidase) to inhibit the synthesis of Hsp70 or Hsp90, which improve the efficacy of low-hyperthermia PTT [24, 25]. In addition, some groups have reported to construct nanoparticles to deliver typical inhibitor 17-allylamino-17-demethoxygeldanamycin (17-AAG), which decrease the PTT induced expression of Hsp to achieve enhanced mild-temperature PTT efficiency [26]. Therefore, co-delivery of Hsp inhibitor with NIR photosensitizers may be a potential strategy to overcome tumor thermoresistance for maximizing PTT. Gambogic acid (GA) is a natural anti-cancer drug, which could inhibit Hsp90 to increase sensitivity of PTT [27–29]. Nonetheless, the clinical application is restricted by the poor water solubility and limited bioavailability. Therefore, it is urgent to develop a novel strategy to co-deliver MnO_2_ and GA, which reverse hypoxia for PDT and reduce Hsp90 expression for PTT.

Human serum albumin is the most abundant plasma protein and has great potential as a safety natural carrier to deliver therapeutic agents [30, 31]. Due to its biocompatibility, low cost and easy preparation, albumin protein-based nanoparticles have been applied for various disease treatments, such as retinal ischemia, liver fibrosis, rheumatoid arthritis, and cancer [32–34]. Owing to the specific binding affinity between albumin and overexpress receptors of cancer cells, the formed albumin nanoparticles could accumulate at tumor site via enhanced permeability and retention (EPR) effect and receptor-mediated endocytosis [35]. Abraxane, is the albumin nanoparticles to deliver paclitaxel, which has been approved in clinical application of cancer treatment by FDA [36, 37]. Besides delivery of chemotherapeutic drugs, photosensitizers such as chlorin e6 and Indocyanine green have been encapsulated into albumin nanoparticles for PDT of cancer [38, 39]. Thanks to the 3D structure of albumin with hydrophobic and hydrophilic domains, together with abundant active groups, albumin is able to deliver various drug molecules and metal ions for combination therapy [40]. Therefore, human serum albumin is suitable as drug carrier for cancer treatment.

**Fig. 1:**
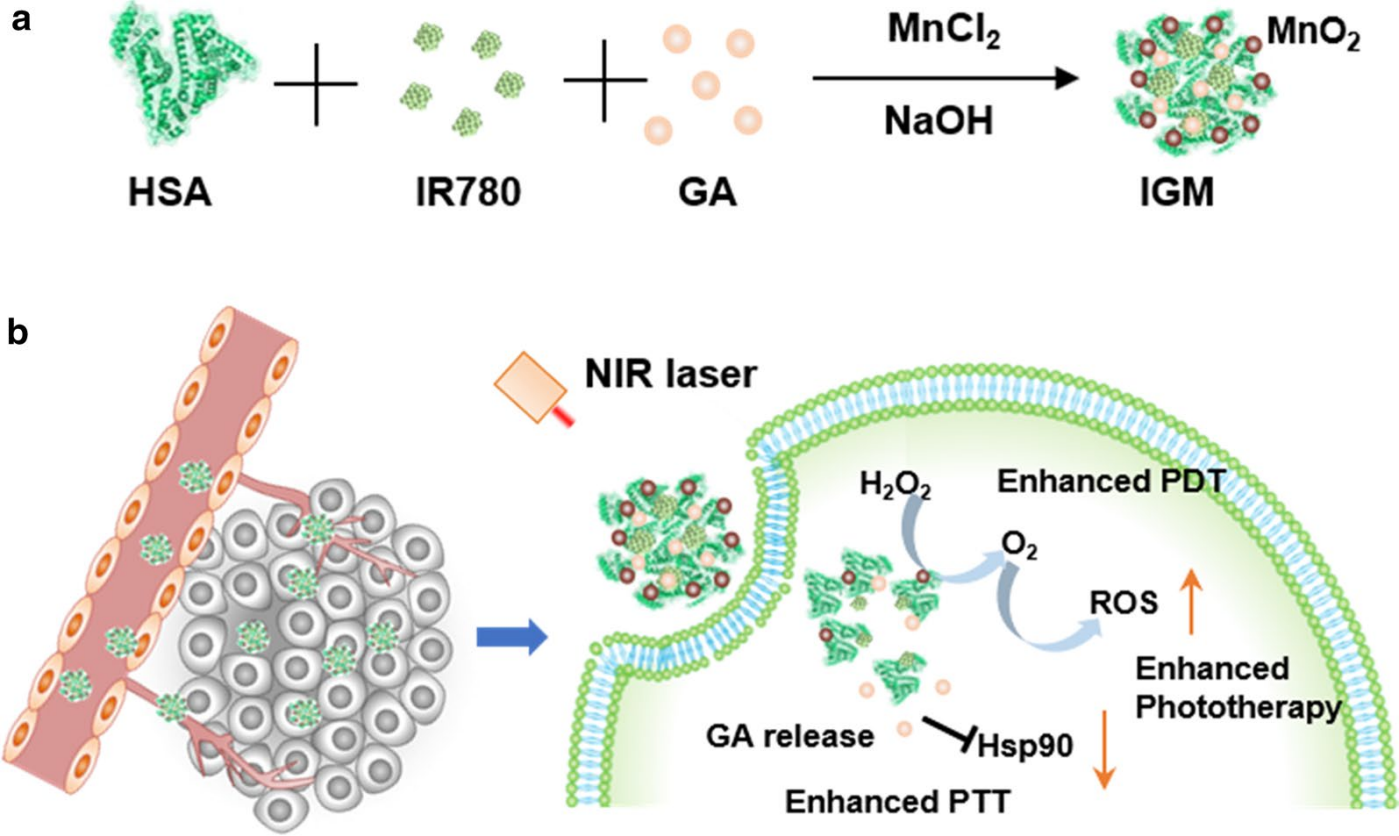
Illustration of IGM for enhanced phototherapy. **a** Construction of IGM via self-assembled method. **b** After IGM accumulation at tumor site, IGM catalyzes H_2_O_2_ to reverse hypoxia for PDT enhancement and bind with Hsp90 to recover sensitivity of thermal effect for PTT enhancement

Taking all advantages into consideration, in this study, we developed self-assembled albumin nanoparticles (IGM) to encapsulate IR780 (NIR photosensitizer) and GA (Hsp90 inhibitor), which were further combined with MnO_2_ in the surface of nanoparticles with one-pot method (Fig. 1a). Due to the EPR effect, after intravenous injected into tumor bearing mice, IGM could accumulate at tumor site and catalyze the overexpress of H_2_O_2_ to generate O_2_, which could reverse hypoxic tumor microenvironment. With NIR laser irradiation, IGM could produce more singlet oxygen (^1^O_2_) or ROS to induce cell death via enhanced PDT. In addition, the GA could bind with Hsp90 to decrease its expression, which could increase sensitivity of PTT and reverse thermoresistance to enhance PTT therapeutic efficacy in vitro and in vivo (Fig. 1b). This study provides a simple strategy to form albumin nanoparticles, which could enhance phototherapy of NIR photosensitizers by both enhanced PDT and PTT for clinical cancer treatment.

## Materials and methods

### Materials

HSA solution was obtained from Octapharma. Gambogic acid (GA) and IR780 were purchased from Aladdin Industrial Corporation (Shanghai, China). H_2_DCFDA and Cell Counting Kit-8 (CCK-8) was purchased from Beyotime Institute of Biotechnology (China). Singlet Oxygen Sensor Green (SOSG) was obtained from Thermo Fisher Scientific. Cytotoxicity ROS-ID Hypoxia/Oxidative Stress Detection Kit was obtained from Enzo Life Sciences. The other chemicals were used as obtained commercially without any further purification.

### Preparation of IGM

The methanol solution (200 μl, IR780, 5 mg/ml and GA 4 mg/ml) was slowly dropped into HSA solution (2 ml, 20 mg/ml) and stirring for 30 min. After that, 100 μl of MnCl_2_ solution (20.2 mg/ml) was added and adjust pH to 9.0 with NaOH (1.0 M) [41, 42]. Then, the solution was stirred at 37 °C for another 2 h and dialyzed for 24 h to obtain IGM nanoparticles. They were stored at 4 °C for further experiments.

### Characteristic of IGM

The concentration of IR780 and GA was determined by the UV–vis absorbance at 790 nm and 350 nm, respectively. The concentration of MnO_2_ was determined by ICP. The size and storage stability of IGM was determined by DLS (Malvern, UK). The structure and morphology were determined by transmission electron microscope (TEM).

### Drug release of IGM

IGM (1 ml, GA, 80 μg/ml) were dialyzed against 10 ml PBS (pH 6.0 and 7.4) with 0.1% (w/v) Tween-80. At different time intervals, dialysis solution (1 ml) was obtained to determine by UV–vis spectrophotometer and then 1 ml PBS was added to keep dialysis system. Also, the NIR triggered GA release was evaluated in PBS (pH = 6.0) after dialyzed 12 h. After with NIR irradiation (808 nm, 0.8 W/cm^2^) for 2 min, the determination was similar as described.

### Oxygen generation and photodynamic effect of IGM

The property of oxygen generation of IGM with addition of H_2_O_2_ was evaluated by Oxygen probe. In detailed, IG and different concentrations of IGM (0.5 ml, MnO_2_, 40 and 80 μg/ml) was added to H_2_O_2_ (3.5 ml, 1 mM) and the oxygen concentration was recorded within 4 min. SOSG (Ex/Em = 490/525 nm) was used to determine the generation of ^1^O_2_, which was oxidized with ^1^O_2_ to emit green fluorescence. Different samples (100 μl) was mixed with SOSG (20 μl, 50 μM) and irradiated with NIR laser (808 nm, 0.8 W/cm^2^). The fluorescence was recorded by fluorescence microplate reader.

### Photothermal effect of IGM

Different concentrations of IGM (IR780, 5, 10, and 20 μg/ml) was irradiated under 808 nm NIR laser at a power density of 0.8 W/cm^2^ within 2 min. The water was set as control. The increased temperature was recorded by thermometer and visual IR thermometer (FLIR Tools). In addition, the photothermal effect of IGM under different power density (0.8 and 1.6 W/cm^2^) was also determined by described method.

The photothermal conversion efficiency was calculated according to the previous literature [43].

$$\eta = \frac{{{\text{hS(T}}_{\hbox{max} } - T_{surr} )- Q_{dis} }}{{{\text{I}}(1 - 10^{ - A808} )}}$$

$$\tau_{S} = \frac{{m_{D} c_{D} }}{hS}$$

η is the photothermal conversion efficiency. T_max_ and T_surr_ means the equilibrium and surround temperature for solution, respectively. *I* represent the power of laser (0.8 W/cm^2^) and A is the absorbance of IR780 at 808 nm. m_D_ is the mass of water (0.3 g) and c_D_ is the heat capacity of water (4.2 J/g).

### Intracellular relief of hypoxia

The 4T1 cells were cultured in 96-well plate at density of 8000 per well and incubated for 24 h. After cells incubated with the mixture solution of Hypoxia Detection Reagent (1 μM and Ex/Em = 596/670 nm) and IGM or IG (MnO_2_ 20 and 50 μg/ml), the upper medium was covered by liquid paraffin for 3 h to create hypoxic condition (3–5%). Then, the cells were washed with PBS, fixed with 4% paraformaldehyde, and stained with DAPI. At last, the fluorescence of hypoxia in tumor cells were captured by fluorescence microscope (Nikon, Japan) and calculated by ImageJ software.

### Intracellular ROS generation of IGM

H_2_DCFDA (Ex/Em = 495/529 nm) was introduced to detect intracellular ROS. The 4T1 cells were incubated with IG, IGM (IR780 8 μg/ml and MnO_2_ 40 μg/ml) and H_2_DCFDA for 3 h. After washed with PBS, the 4T1 cells were performed with NIR laser irradiation (808 nm, 0.8 W/cm^2^) for 1 min. Then, the cells were stained with DAPI and the fluorescence was obtained by fluorescence microscope (Nikon, Japan). Also, the intracellular ROS generation was further analyzed by flow cytometer (ACEA). The protocol was similar as described above.

### Cytotoxicity of IGM and western blot of HSP90 in vitro

The 4T1 cells (8000 cells per well) were seeded in 96-well plates and incubated with different concentrations of IG, IGM and IM for 3 h. After that, tumor cells were washed twice with PBS and followed with 1 min NIR laser irradiation (808 nm, 0.8 W/cm^2^). The cell viability was determined by CCK-8 assay according to the standard protocol. In addition, the Calcein AM/PI staining was also performed to evaluate anti-tumor property of IGM in vitro. The protocol was similar as described above and the fluorescence images were captured by fluorescence microscope (Nikon, Japan). For intracellular western blotting expression of HSP90, the 4T1 cells were seeded into a 6-well plates with a density of 2 × 10^4^ cells/cm^2^ and incubated for 24 h. Then, IM and IGM (IR780 10 μg/ml and MnO_2_ 40 μg/ml) were added and incubated with 3 h. Then, the cells were collected and irradiated with NIR laser irradiation (808 nm, 0.8 W/cm^2^) for 1 min. After that, the cells were lysed by and the total protein was determined by BCA protein assay kit (Beyotime). Then, the protein was performed with 12% SDS-PAGE gel and transferred to a polyvinylidenedi-fluoride (PVDF). Then the western blot of HSP90 (Abcam) was determined by HSP90 primary antibody according to standard protocols.

### Time-dependent biodistribution of IGM in vivo

When tumor reached about 200 mm^3^, IGM (IR780 112 μg/ml) was intravenously injected into 4T1 tumor bearing mice. At different time point (2, 4, 8, 12, 24, and 48 h), the fluorescence of IR780 was capture by CRI maestro system. At 48 h post-injection, the organs and tumor were collected and perform fluorescence images of IGM. The semi-quantitative biodistribution analysis was calculated by fluorescence signals.

### Photothermal effect and reverse hypoxia of IGM in vivo

After 24 h post intravenous injection of saline and IGM (IR780 120 μg/ml and MnO_2_ 360 μg/ml), the tumor was collected and stained with HIF-1α to evaluate the relief hypoxia of IGM. In addition, the NIR laser were irradiated at tumor site and the temperature were recorded by a visual IR thermometer within 2 min.

### Anti-tumor effect in vivo

When tumor size reached about 100 mm^3^, 4T1 tumor bearing mice were randomly divided into five groups (six mice in each group), including Saline, IGM, IGM with NIR, IG with NIR, and IM with NIR (IR780 134 μg/ml, GA 120 μg/ml, and MnO_2_ 410 μg/ml). After 24 h post intravenous injection, the groups with NIR laser (808 nm, 0.8 W/cm^2^) were performed with irradiation for 2 min. The tumor size and body weight were recorded every 2 days. The volume was calculated based on the formula: V = width^2^ * length/2. Relative tumor volumes were recorded as V/V_0_. After finished treatment at day14, the tumors were collected and weighed. In addition, H&E, TUNEL, and HSP90 staining were performed at day3 to further evaluate the therapeutic efficacy of IGM.

### Biosafety of IGM

For biosafety evaluation, BALB/C male mice were intravenously injected with saline and IGM (IR780 134 μg/ml, GA 120 μg/ml, and MnO_2_ 410 μg/ml). After 24 h post injection, blood was collected to determine AST, ALT, CREA and BUN, which respected liver and kidney function. Major organs were collected to preform H&E staining.

## Results

### Preparation and characteristic of IGM

**Fig. 2:**
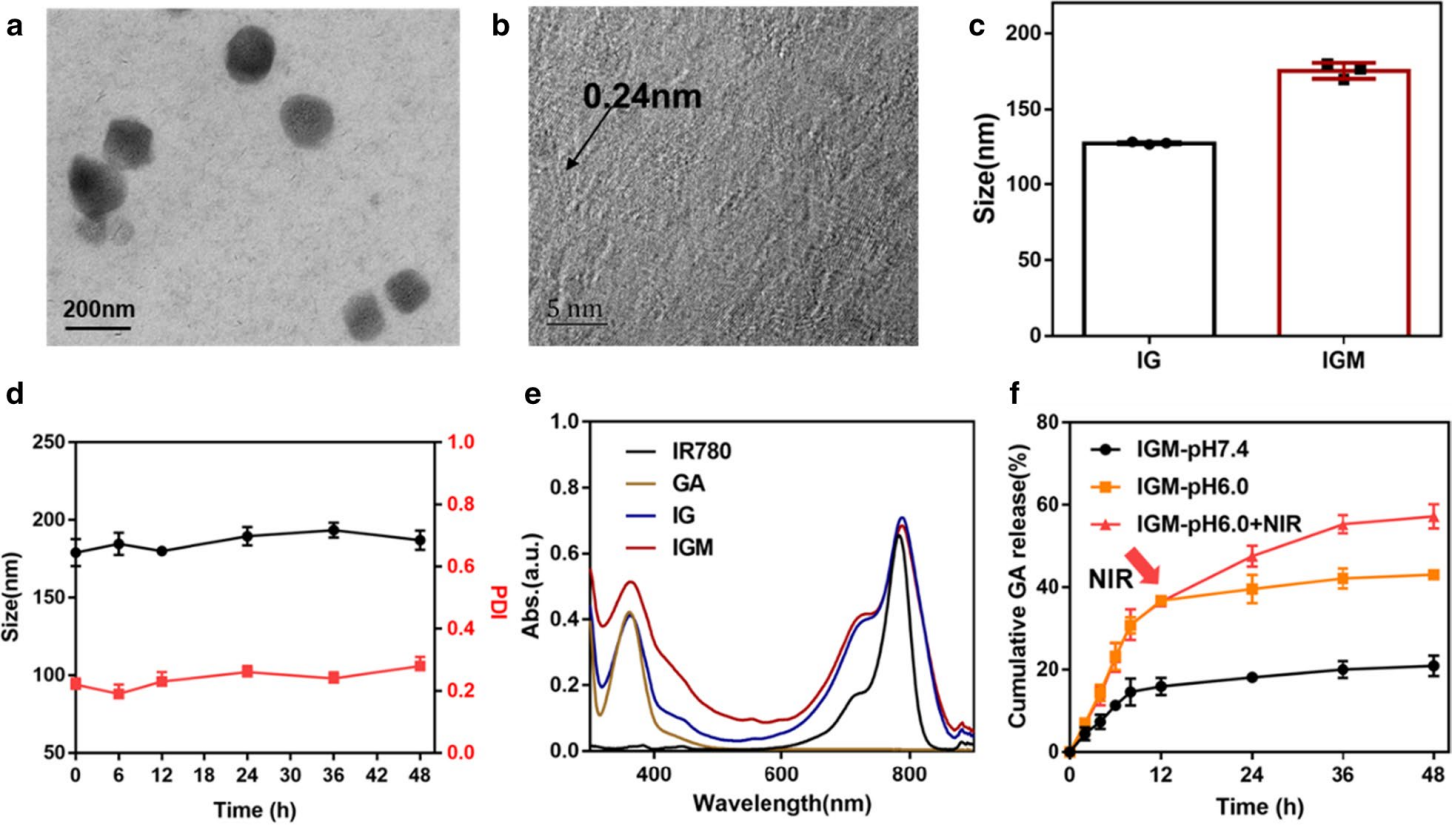
Characteristics of IGM. **a**, **b** TEM (Scale bar = 200 nm) and HRTEM (Scale bare = 5 nm) image of IGM. **c** Size distribution of IG and IGM. **d** Stability of IGM within 48 h. **e** UV absorbance spectrum of IR780, GA, and IGM. **f** In vitro release profile of IGM in different PBS buffer (pH 6.0 and 7.4) with or without laser irradiation (808 nm, 0.8 W/cm^2^, 1 min). Data are expressed as mean ± SD (n = 3)

The IGM nanoparticles were synthesized by one-pot method. IR780 and GA were the highly hydrophobic drugs to induce self-assembly of HSA, due to hydrophobic interactions between drug molecules and the hydrophobic domain of HSA. After mixed with MnCl_2_, Mn^2+^ would be anchored to active groups of HSA to form IG-Mn complex due to electronic interaction. With pH adjustment of NaOH, the Mn^2+^ would generate MnO_2_ dropped in IG albumin nanoparticles. Transmission electron microscopy (TEM) images showed the spherical morphology of IGM (Fig. 2a). The structure of MnO_2_ in IGM was determined by high resolution TEM (HRTEM), which showed the lattice fringes with a lattice distance of 0.24 nm (Fig. 2b). The hydrodynamic diameter of IGM was about 188 nm determined by DLS, which is larger than that of IG (140 nm). This may be due to the generation of MnO2 in the IG nanoparticles (Fig. 2c). In addition, as shown in Fig. 2d, there was no significant changes of size with 48 h during the storage at 4 °C. The zeta potential of IGM was about -24mv, which is decreased than that of IG (-18mv), as shown in Additional file 1: Figure S1. X-ray photoelectron spectroscopy (XPS) was employed to evaluate the chemical compositions of IGM. As shown in Additional file 1: Figure S2, XPS survey and Mn2p spectrum showed that MnO_2_ was effectively dropped to IG nanoparticles. The characteristic absorbance of IR780 and GA was 790 and 350, which was observed in IGM (Fig. 2e). Meanwhile, fourier transform infrared (FTIR) spectra was applied to further confirm the successful encapsulation of IR780 and GA. As shown in Additional file 1: Figure S3, the main characteristic peaks of IR780 and GA was appeared in the formed IGM nanoparticles. The standard line of GA was shown in Additional file 1: Figure S4, determined by UV absorbance of 350 nm. The concentration of IR780 and GA was calculated based on absorbance and the loading content of IR780 and GA was 4.2% and 2.6%, respectively. All these results indicated that IR780, GA and MnO_2_ were successfully encapsulated in HSA to form IGM nanoparticles.

### Main property of IGM

The GA release was performed in different PBS buffer solution (pH 6.0 and 7.4). As shown in Fig. 2f, within 12 h, GA was released quickly in both pH 6.0 and 7.4 of buffer solution. But, the release of GA reached approximately 40% in pH 6.0 buffer solution, which is nearly twofold of that in pH 7.4 buffer solution (17.6%). Then, after irradiated with NIR laser (808 nm, 0.8 W/cm^2^), the release of GA was significantly increased compared to that without laser irradiation in pH 6.0 buffer solution. In addition, the size distribution of IGM in different temperature was also evaluated by DLS. As shown in Additional file 1: Figure S5, with increased temperature from 37 to 50 °C, the size of IGM was decreased from 170 nm to 90 nm. The result indicated that with PTT effect of IGM, IGM could be disassembled to release GA. All these results indicated that IGM was stable in blood circulation with little leakage and could release quickly under acid tumor microenvironment and NIR laser irradiation.

**Fig. 3:**
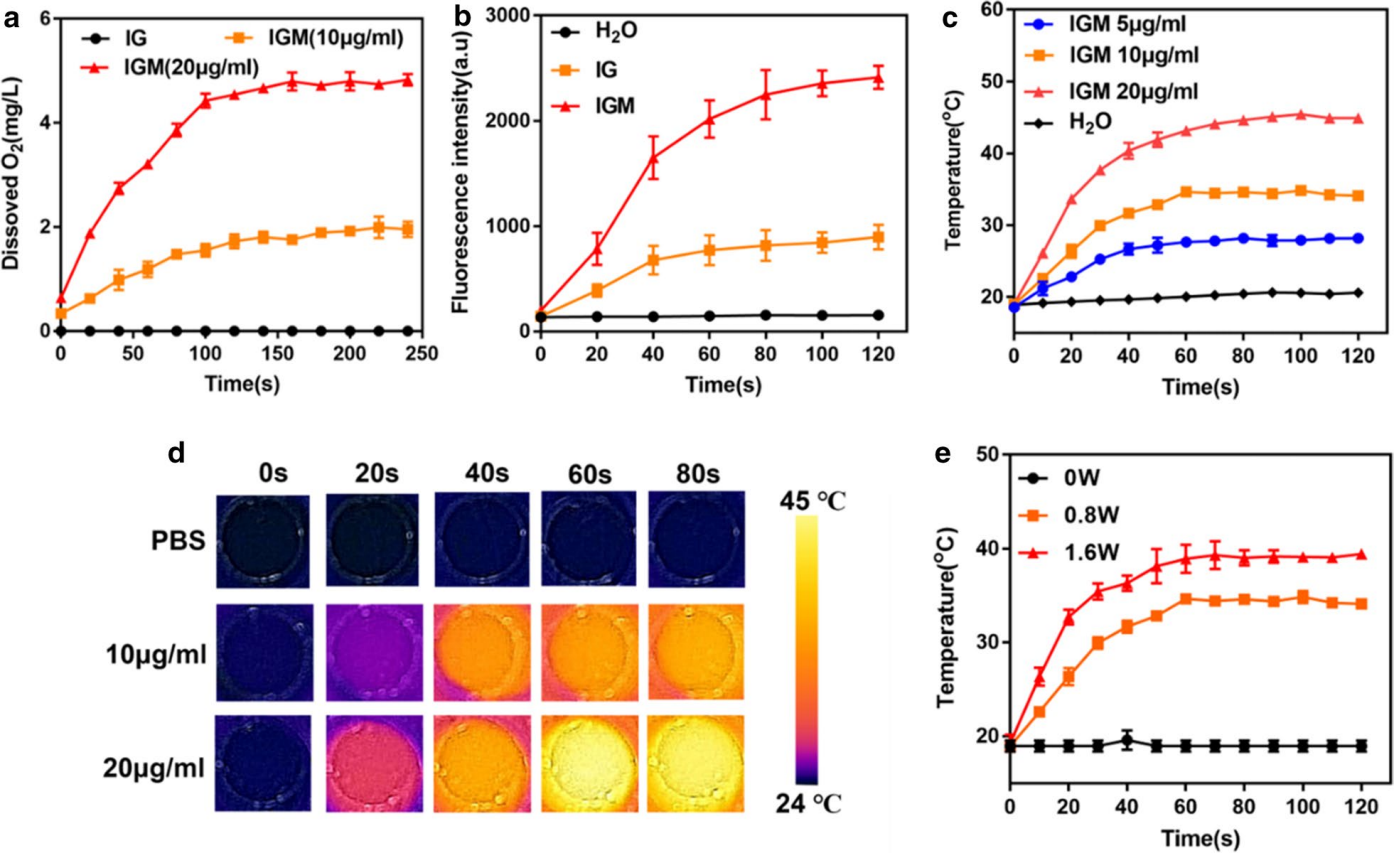
Main properties of IGM in vitro. **a** Oxygen generation of different concentrations of IGM (MnO_2_ 10 and 20 μg/ml) after addition with H_2_O_2_ (1 mM). **b** Singlet oxygen generation of IG and IGM after addition with H_2_O_2_ (1 mM). **c** Photothermal heating curves and **d** photothermal images of IGM at various concentrations under laser irradiation (808 nm, 0.8 W/cm^2^). **e** The increased temperature profiles of IGM (IR780 10 μg/ml) with laser irradiation (808 nm, 0.8 and 1.6 W/cm^2^). Data are expressed as mean ± SD (n = 3)

The MnO_2_ could catalyze H_2_O_2_ to produce O_2_, which has been widely used to reverse hypoxia of tumor [44]. The catalyze property to generate O_2_ was then evaluate in Fig. 3a. With addition of IG without MnO_2_ into H_2_O_2_ solution (1 mM), there was no oxygen generation. While, different concentration of IGM added into H_2_O_2_ solution (1 mM), the generation of O_2_ was increased with time and concentration of MnO_2_ in IGM. IR780 was applied as a photosensitizer to produce ^1^O_2_ for PDT of cancer treatment. SOSG was specific fluorescence probe to be oxidized by ^1^O_2_ [45]. As shown in Fig. 3b, the generation of ^1^O_2_ in IG and IGM was increased with irradiation time. Due to the catalyzation of H_2_O_2_ to produce O_2_, the ^1^O_2_ in IGM was about 2.5-fold than that in IG. These results indicated that our formed IGM could release O_2_ to generate more ^1^O_2_ for PDT enhancement.

Apart from the photodynamic property of IR780, it could also absorb laser energy to produce heat as photothermal effect for cancer treatment. As shown in Fig. 3c, compared with control group (water), the temperature of IGM was went up quickly with increasing concentration of IR780, indicating a concentration-dependent manner after NIR laser irradiation. The increased temperature of IGM (IR780 20 μg/ml) was 24.2 °C, which is nearly twofold than that of IGM (IR780 10 μg/ml). The photothermal effect was also evaluated by IR thermal images with increased irradiation time (Fig. 3d), which is similar as that determined by thermometer. In addition, the increased temperature of IGM with different laser power was also evaluated, as shown in Fig. 3e. The temperature was significantly increased in both power laser and the temperature irradiated with 1.6 W/cm^2^ was higher than that irradiated with 0.8 W/cm^2^ (△T 20 vs 14 °C). According to the formula, the conversion efficiency of albumin nanoparticles of IR780 was calculated about 29.1%, which is similar as that reported of IR780 nanoparticles in previous studies [46]. As that IR780 can produce heat, so the stability of IR780 in different temperature was evaluated. As shown in Additional file 1: Figure S6, the absorbance of IR780 showed no significant changes in different temperature. All these results indicated that after irradiated with NIR laser, IGM could catalyze H_2_O_2_ to enhance singlet oxygen generation for enhanced PDT and produce heat for PTT.

### Intracellular hypoxia and ROS detection

**Fig. 4:**
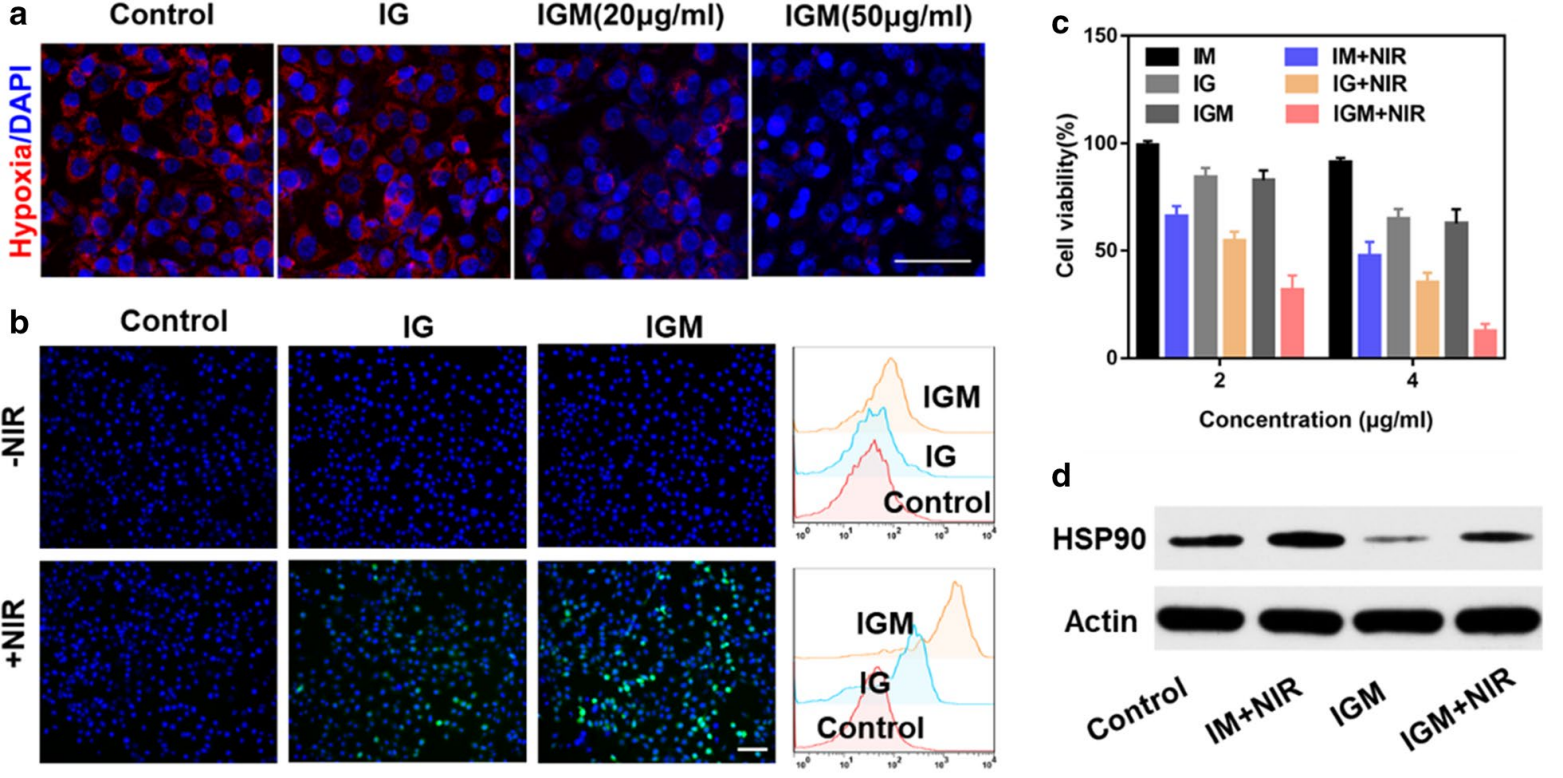
In vitro anti-tumor therapy of IGM. **a** Representative fluorescence images of hypoxia (red) in 4T1 tumor cells after various treatments. **b** Fluorescence images and Flow cytometry quantitative analysis of ROS generation stained with DCFH-DA (green) and the nucleus stained with DAPI (blue) after different treatments. Scale bar is 100 μm. **c** Tumor cell viability after various treatments **d** Western blot of HSP90 expression in different treatments. Data are expressed as mean ± SD (n = 3)

The therapeutic efficacy of PDT was restricted by hypoxic tumor microenvironment. As MnO_2_ could catalyze H_2_O_2_ to produce O_2_, we evaluated the relief hypoxia of IGM in 4T1 tumor cells. The hypoxia of tumor cells was created by covered liquid paraffin. As shown in Fig. 4a and Additional file 1: Figure S7, after incubation with different concentrations of IGM, the cells showed slight red fluorescence, compared with control and IG groups, which showed bright red fluorescence. In addition, with increased concentration of MnO_2_, the red fluorescence was significantly decreased with and no obvious red fluorescence was observed in high concentration of IGM. These results indicated that in hypoxia condition, the MnO_2_ in IGM could produce oxygen to relive hypoxia for further PDT enhancement.

The ROS generation was detected by H_2_DCFDA fluorescence probe, which was excited by ROS to emit green fluorescence. As shown in Fig. 4b, without NIR laser irradiation, the 4T1 cells showed slight green fluorescence. While, after irradiated with 1 min, the 4T1 cells of IG also showed green fluorescence due to ROS generation of PDT. But, the cells of IGM showed bright green fluorescence than that of IG and control group, because of the MnO_2_ of IGM produce O_2_ to enhance ROS generation for PDT. In order to further evaluate ROS generation, DCF staining were determined by quantitative flow cytometry analysis. The trends were similar as that observed by fluorescence images. All these results indicated that IGM could reverse hypoxia to enhance ROS generation for enhanced PDT.

### Cell viability of IGM

The combination antitumor activity of enhanced PDT and PTT was evaluated in 4T1 cells by CCK-8 assay and PI/calcein-AM staining. As shown in Fig. 4c, without laser irradiation, the cell viability was decreased with the concentration of GA, which may be due to the anti-tumor effect of GA as chemotherapy drug. After irradiated with NIR for 1 min, all these groups showed antitumor therapeutic efficacy. The cells of IGM (IR780 4 μg/ml) only maintained approximately 15% viability, which was 2.5 times as low as IG with laser irradiation (40%). This indicated that MnO_2_ could increase ROS generation to enhance PDT for cancer therapy. Also, compared with IM, the cell viability was significantly decreased, due to the contribution of GA. In addition, the cell toxicity of IGM in HUVEC cells was also evaluated. As shown in Additional file 1: Figure S8, after incubation of albumin nanoparticles with HUVEC cells and 4T1 tumor cells, without laser irradiation, IGM showed little toxicity to HUVEC cells, but significantly killed 4T1 cell. This may be due to high affinity of HSA with the overexpressed of gp60 in tumor cells [47]. With the increase concentration of IR780, the toxicity to 4T1 cells was increased and also induced HUVEC cells apoptosis. This may be due to the higher toxicity of GA.

It has been reported that GA could bind with HSP90, which can increase the sensitivity of photothermal effect [48]. In order to confirm the enhanced therapeutic mechanism of IGM, western blotting was further used to study the expression of HSP90 in 4T1 tumor cells after various treatments. As shown in Fig. 4d, compared with control group, irradiated with NIR laser, IM showed increased expression of HSP90, which is also higher than that of IGM with NIR, due to the binding effect of GA to HSP90. In addition, the 4T1 cells treated with IGM showed significantly decreased expression of HSP90 than all other groups, which is further confirmed that GA could reduce HSP90 expression. These results were also evaluated by immunofluorescence staining of HSP90 in 4T1 cells. As shown in Additional file 1: Figure S9, the red fluorescence of 4T1 cells in group IGM with laser is decreased than that of IM with NIR. All these results indicated that GA in IGM could enhance sensitivity of tumor cells to PTT, which could increase antitumor activity of combined enhanced PDT and PTT.

### Biodistribution and photothermal effect of IGM in vivo

**Fig. 5:**
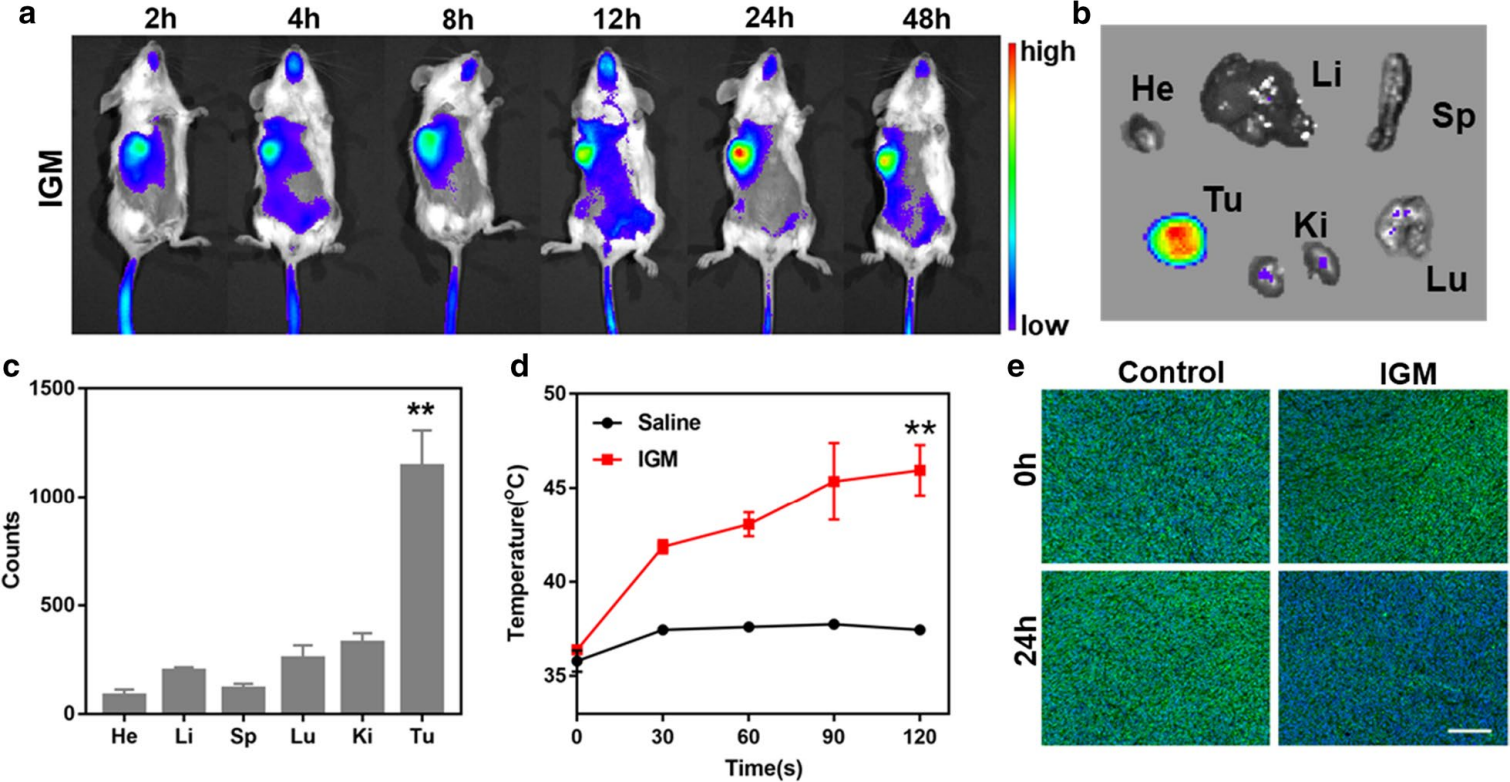
Biodistribution of IGM in vivo. **a** Dynamic fluorescence images of IGM (IR780, 120 μg/ml) in 4T1 tumor-bearing mice at different time points after intravenous injection. **b** Ex vivo NIR fluorescence images and **c** quantitation of major organs and tumor at 48 h after IGM treatment (n = 3, **P<0.01, tumor vs. other organs). **d** The increased temperature of tumor at 24 h post injection with laser irradiation(n = 3, **P<0.01, IGM vs. saline). **e** Immunofluorescence images of tumor slices stained by HIF-1α after intravenous injection of IGM at 0 h and 24 h. Data are expressed as mean ± SD (n = 3)

Due to the EPR effect of tumor, after intravenous injection of IGM, 4T1 tumor bearing mice were imaged by NIR fluorescence imaging system. As shown in Fig. 5a, the accumulation of IGM at tumor site was enhanced with time during the period of 2–48 h post injection. The maximum fluorescence of IR780 in tumor was observed at 24 h post injection. Afterward, the biodistribution of IGM in major organs and tumor was evaluated at 48 h post injection. The tumor ex vivo showed significant fluorescence signals of IR780 than other organs (Fig. 5b, c), which indicated that IGM could accumulate at tumor site in vivo for cancer treatment.

As confirmed that IGM could act as photothermal agent for PTT, after 24 h post injection, upon NIR laser irradiation, the increased temperature at tumor site was monitored by an infrared thermal. As shown in Fig. 5d and Additional file 1: Figure S3, the tumor in control group showed slightly increase in temperature (only increased 2 °C), while the temperature of tumor in IGM increased quickly about 5 °C in the first 30 s and increased to 10 °C with laser time of 2 min. These results indicated that IGM could perform PTT in vivo. Also, in vitro, IGM could generate O_2_ to reverse hypoxia. The immunofluorescence of HIF-1α at tumor slices was evaluated after 24 h post intravenous injection of IGM. As shown in Fig. 5e and Additional file 1: Figure S10, the tumor in control group showed bright green fluorescence, which is higher than that of tumors accumulated with IGM (1862 vs. 473). These results confirmed that IGM could be also reverse hypoxia of tumor microenvironment in vivo for PDT enhancement.

### Anti-tumor and biosafety of IGM in vivo

**Fig. 6:**
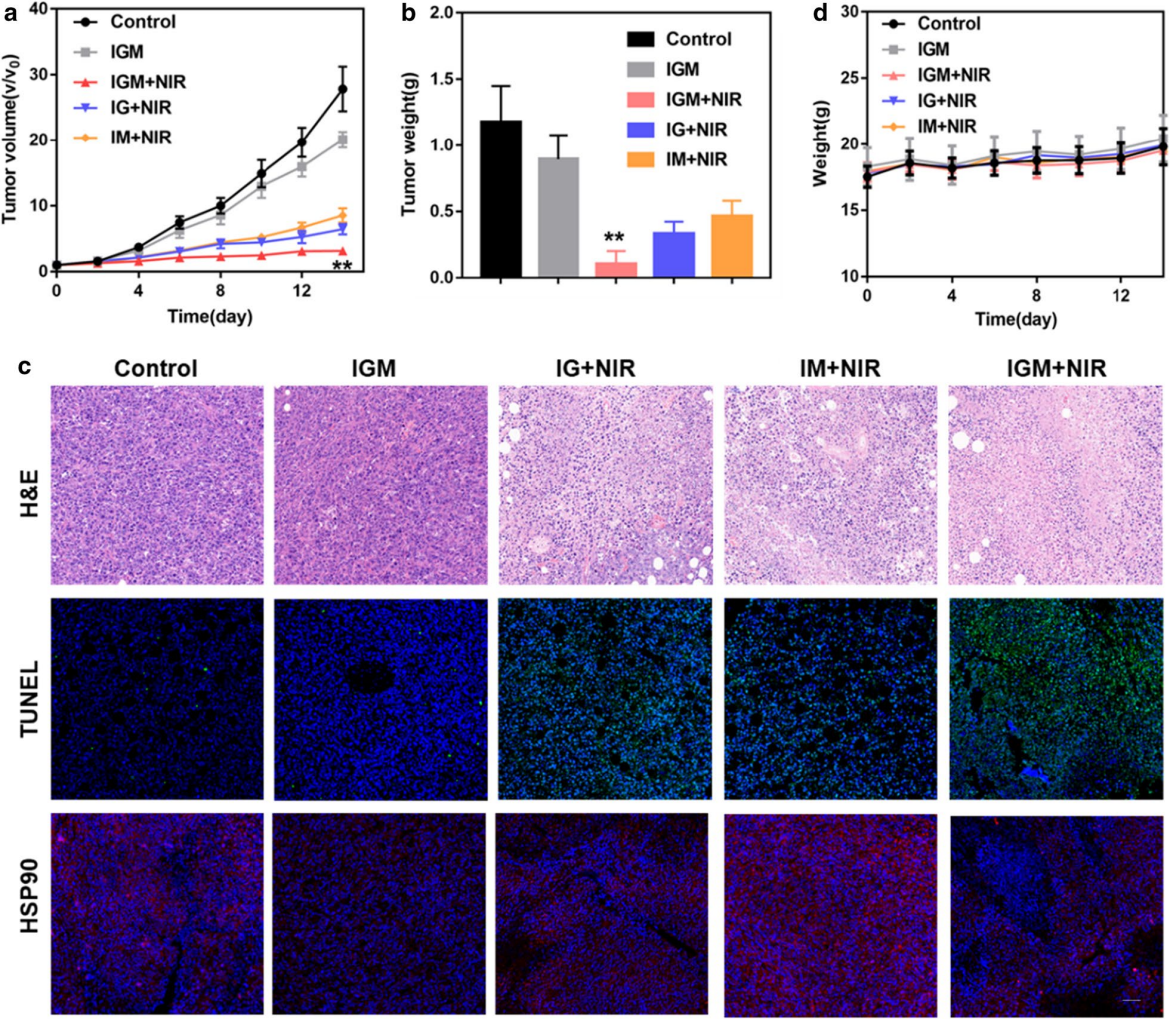
Anti-tumor effect of IGM in vivo. **a** Time-dependent tumor growth curves in 4T1 tumor after various treatments with intravenous injection (n = 6, **P<0.01, IGM + NIR vs. other groups). **b** Tumor weight at the end of treatment (n = 6, **P<0.01, IGM + NIR vs. other groups). **c** H&E, TUNEL, and HSP-90 staining of tumors at day 3 after various treatments (Scale bar = 100 μm). **d** Body weight of mice in different groups during therapy period. Data are expressed as mean ± SD (n = 6)

Encouraged by the accumulation, photothermal effect and relief of hypoxia, the antitumor therapeutic efficacy of IGM was evaluated in 4T1 tumor bearing mice in vivo. When the tumor reached 100 mm^3^, they were randomly divided into 5 groups, including control, IM with laser, IG with laser, IGM, and IGM with laser. As shown in Fig. 6a, compared with control group, IGM without laser showed slight inhibition, due to the therapeutic efficacy of GA. After irradiation with NIR laser, IGM showed significant tumor inhibition compared with other groups (IG or IM with laser), due to the binding effect of GA for PTT enhancement and relief hypoxia of MnO_2_ for PDT enhancement. In addition, IG or IM with laser also showed tumor inhibition than IGM without NIR, because of the activation of IR780 for phototherapy of cancer. After various treatment at day 14, the tumor was collected to weigh, as shown in Fig. 6b. The tumor inhibition of IGM with laser was approximately 91%, which is higher than that in IG with laser (72%) and IM with laser (61%). These results indicated that IGM could significantly inhibit tumor growth through combination of enhanced PDT and PTT.

In order to further confirm the therapeutic efficacy, the cell apoptosis was evaluated by H&E and TUNEL fluorescence staining. As shown in Fig. 6c, the nuclear of tumor cells in IGM with NIR was significantly broken and the green fluorescence was higher than that in other treated groups. These results were consistent with the tumor inhibition study. The collected tumors were further prepared to tissue sections for the immunofluorescence staining of HSP90, which confirmed the therapeutic enhancement of PTT by the inhibition of thermo-resistance. As shown in Fig. 6c and Additional file 1: Figure S11, with NIR laser irradiation, compared with control and IM with NIR group, the fluorescence of HSP90 was significantly reduced in IGM (). The IGM and IG with NIR group also reduced HSP90 expression. All these results indicated that GA could reduce HSP90 expression for PTT enhancement.

**Fig. 7:**
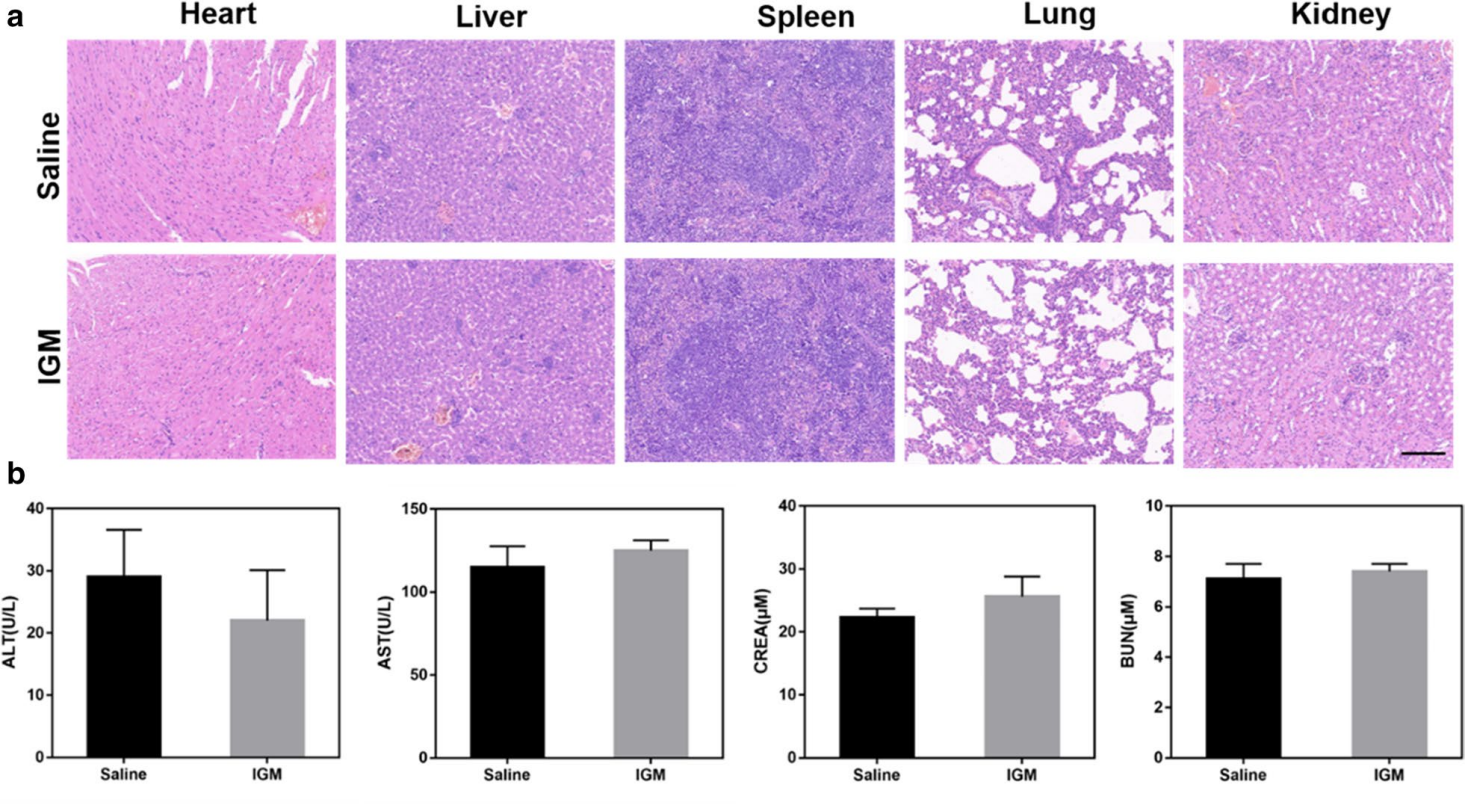
Evaluation safety of IGM in vivo. **a** H&E staining of major organs and **b** Serum biochemistry of liver and kidney function markers after intravenous injection of IGM. Data are expressed as mean ± SD (n = 3)

In order to evaluate the biosafety of IGM, the body weight was determined during the treatment. As shown in Fig. 6d, there was no obvious changes of mice weight. In addition, the major organs were collected after intravenous injection of saline and IGM. As shown in Fig. 7a, there was no obvious damage of IGM observed in the H&E staining of major organs. Furthermore, two hepatic function parameters (AST and ALT) and two kidney function parameters (CREA and BUN) of IGM group showed no significant difference to that of control group, which was in good agreement with the H&E staining (Fig. 7b). These results showed that IGM was safe for antitumor therapy in vivo.

## Discussion and conclusion

Phototherapy is increasingly recognized to be a promising strategy for primary cancer treatment, which shows high selectivity and effectivity. It applied phototherapy agents to convert light energy to generate heat as PTT and produce ROS as PDT [49]. However, two major obstacles of the tumor limited its therapeutic efficacy in cancer treatment. The first one is the hypoxia tumor microenvironment, which restricts the ROS generation, especially ^1^O_2_ [14]. The second one is the overexpression of HSP accompanied with increased temperature, which reduces the therapeutic efficacy and lead to high chances of recurrence [25]. Therefore, effective strategies to overcome hypoxia and reduce expression of HSP are urgent to enhance phototherapy of cancer. We thus constructed albumin nanoparticles dropped with MnO_2_ to co-deliver GA and IR780 via one-step self-assemble method.

In this study, HSA was chosen as drug carrier due to the abundant active groups and hydrophobic domains, which could encapsulate IR780 and GA [50]. In recent years. gold nanostructures and two-dimensional nanomaterials have also been attracted great attention as photothermal agents and drug carrier for combination therapy of cancer [51]. Control the aspect ratio of gold could achieve a sensitive response to NIR light with high tumor penetration [52]. In addition, other therapeutic drugs such as anticancer drugs and nucleic acid could conjugate or absorb in the surface of gold nanostructure to inhibit tumor growth [53]. Compared with gold, the inorganic nanostructures of 2D nanomaterials such as selenium tellurium, 2D black phosphorus, and 2D antimonite nanoparticles showed improved biocompatibility, high surface area for efficient loading of various functional molecules, unique surface chemistry for modification, and inherent optical properties with strong absorbance of NIR light for PTT [54–56]. However, the long-term safety concerns, interaction with immune system and excretion of these nanomaterials are crucial issues for clinical application [57, 58]. HSA is abundant in plasma and has been approved in clinical use. In addition, specific hydrophilic binding sites and the high content of functional groups could make it suitable to deliver photosensitizer and chemo drugs for combination therapy [59].

The release of GA was increased in the acid tumor microenvironment and also triggered with the laser irradiation (Fig. 2f). This may be due to the degradation of MnO_2_ and disassemble of albumin nanoparticles. GA has been reported as a protentional anti-cancer drug in clinical application, which was shown cell killing and tumor inhibition (Figs. 4c, 6a). It can inhibit expression of HSP90 after PTT treatment (Figs. 4d, 6c). This results indicated that combined GA and phototherapy could overcome tumor thermoresistance for maximizing therapeutic efficacy of PTT. In addition, MnO_2_ was dropped in the albumin nanoparticles to overcome tumor hypoxia (Figs. 3a, 5e). With laser irradiation, the ROS generation of PDT was significantly increased to induce cell apoptosis and inhibit tumor growth (Figs. 3b, 4b, 6c). These results indicated our design could overcome the two major obstacles in phototherapy to increase therapeutic efficacy of cancer. The formed albumin nanoparticles showed no significant toxicity to major organs (Fig. 7), which indicated that the FDA approved HSA drug carrier showed potentials for clinic applications. Also, some risks of the formed nanoparticles need to take into consideration in the further investigation [31, 60]. The first one is that IR780 is not approved and has toxicity which limit its application. The second one is that the potential immunogenicity of HSA may also exist in the clinical application. The third one is that it is urgent to develop highly reproducible methods for large scale production of albumin nanoparticles.

In summary, HSA nanoparticles (IGM) to co-deliver GA, IR780 and MnO_2_ through one-step self-assemble method was developed to enhance phototherapy of cancer. It could catalyze the overexpress of H_2_O_2_ to produce O_2_ and relive hypoxia, thus generating more ROS for PDT enhancement. It could also efficiently generate heat and promote the release of GA. The released GA could bind with HSP90 to decrease the thermo-resistance of cancer cells for PTT enhancement. This work demonstrated a new design to enhance the therapeutic efficacy of NIR photosensitizers via relief hypoxic tumor microenvironment and decrease of HSP90, which are promising for clinical cancer therapy.

## Supplementary information

**Additional file 1: FigS1-FigS3.** The main characteristics (zeta potential, XPS and FT-IR) of IGM nanoparticles. **Fig. S4.** Standard line of GA determined by UV-vis spectrophotometer. **Figs. S5–S6.** The size distribution of IGM and stability of IR780 in different temperature. **Fig. S7.** The mean fluorescence intensity of intracellular hypoxia. **Fig. S8.** The cell viability of HUVEC and 4T1 cells after incubation with IGM. **Fig. S9.** The immunofluorescence staining and mean fluorescence intensity of Hsp90 in 4T1 cells after various treatments. **Fig. S10.** IR thermal images of tumor bearing mice with NIR laser irradiation. **Fig. S11.** The mean fluorescence of hypoxia in tumor after intravenous injection of saline and IGM. **Fig. S12.** The mean fluorescence of Hsp90 in tumor after various treatments.

## Data Availability

All data generated or analyzed during this study are included in this published article.

## References

[1] Xie Z, Fan T, An J, Choi W, Duo Y, Ge Y, Zhang B, Nie G, Xie N, Zheng TJ. Emerging combination strategies with phototherapy in cancer nanomedicine. 2020. 10.1039/d0cs00215a 32567633

[2] Shi H, Sadler PJ. How promising is phototherapy for cancer? 2020;2:1–3. 10.1038/s41416-020-0926-3 PMC7492227 32587359

[3] Liu Y, Bhattarai P, Dai Z (2019). Chen XJCSR: Photothermal therapy and photoacoustic imaging via nanotheranostics in fighting cancer. Chem Soc Rev.

[4] Wang H, Chang J, Shi M, Pan W, Li N, Tang BJAC (2019). A dual-targeted organic photothermal agent for enhanced photothermal therapy. Angew Chem Int Ed Engl.

[5] Lan M, Zhao S, Liu W, Lee CS, Zhang W, Wang PJ (2019). Photosensitizers for photodynamic therapy. Adv Healthc Mater.

[6] Deng K, Li C, Huang S, Xing B, Jin D, Zeng Q, Hou Z, Lin JJS (2017). Recent progress in near infrared light triggered photodynamic therapy. Small.

[7] Huang L, Li Z, Zhao Y, Yang J, Yang Y, Pendharkar AI, Zhang Y, Kelmar S, Chen L, Wu WJAM (2017). Enhancing photodynamic therapy through resonance energy transfer constructed near-infrared photosensitized nanoparticles. Adv Matter.

[8] Wang K, Zhang Y, Wang J, Yuan A, Sun M, Wu J, Hu YJ. Self-assembled IR780-loaded transferrin nanoparticles as an imaging, targeting and PDT/PTT agent for cancer therapy. 2016, 6:1–11. 10.1038/srep27421 PMC4899881 27263444

[9] Wang H, Li X, Tse BW, Yang H, Thorling CA, Liu Y, Touraud M, Chouane JB, Roberts MS (2018). Indocyanine green-incorporating nanoparticles for cancer theranostics. Theranostics.

[10] Liang P, Huang X, Wang Y, Chen D, Ou C, Zhang Q, Shao J, Huang W, Dong XJ. Tumor-microenvironment-responsive nanoconjugate for synergistic antivascular activity and phototherapy. 2018; 12:11446–11457. 10.1021/acsnano.8b06478 30345740

[11] Li X, Kwon N, Guo T, Liu Z, Yoon JJ (2018). Innovative strategies for hypoxic-tumor photodynamic therapy. Angew Chem Int Ed Engl.

[12] Ren H, Liu J, Su F, Ge S, Yuan A, Dai W, Wu J, Hu YJ. Interfaces: Relighting photosensitizers by synergistic integration of albumin and perfluorocarbon for enhanced photodynamic therapy. ACS Appl Mater Interfaces. 2017;9:3463–73. 10.1021/acsami.6b14885 28067039

[13] Li N, Xu F, Cheng J, Zhang Y, Huang G, Zhu J, Shen X, He DJ (2018). Perfluorocarbon nanocapsules improve hypoxic microenvironment for the tumor ultrasound diagnosis and photodynamic therapy. ..

[14] Lin T, Zhao X, Zhao S, Yu H, Cao W, Chen W, Wei H, Guo HJT. O_2_-generating MnO_2_ nanoparticles for enhanced photodynamic therapy of bladder cancer by ameliorating hypoxia. Theranostics. 2018; 8:990. 10.7150/thno.22465 PMC5817106 29463995

[15] Yang G, Xu L, Chao Y, Xu J, Sun X, Wu Y, Peng R, Liu ZJN. Hollow MnO_2_ as a tumor-microenvironment-responsive biodegradable nano-platform for combination therapy favoring antitumor immune responses. Nat Commun. 2017, 8:1–13. 10.1038/s41467-017-01050-0 PMC5638920 29026068

[16] Hu J-J, Cheng Y-J, Zhang X-Z (2018). Recent advances in nanomaterials for enhanced photothermal therapy of tumors..

[17] Sharma S, Shrivastava N, Rossi F (2019). Thanh NTKJNT: Nanoparticles-based magnetic and photo induced hyperthermia for cancer treatment..

[18] Yuan J, Liu J, Song Q, Wang D, Xie W, Yan H, Zhou J, Wei Y, Sun X, Zhao LJ. Photoinduced mild hyperthermia and synergistic chemotherapy by one-pot-synthesized docetaxel-loaded poly (lactic-co-glycolic acid)/polypyrrole nanocomposites. ACS Appl Mater Interfaces. 2016; 8:24445–54. 10.1021/acsami.6b07669 27565002

[19] Yang Y, Zhu W, Dong Z, Chao Y, Xu L, Chen M, Liu ZJ. 1D coordination polymer nanofibers for low‐temperature photothermal therapy. Adv Matter. 2017; 29:1703588. 10.1002/adma.201703588 28833643

[20] Gao G, Jiang YW, Guo Y, Jia HR, Cheng X, Deng Y, Yu XW, Zhu YX, Guo HY, Sun WJ (2020). Enzyme-mediated tumor starvation and phototherapy enhance mild-temperature photothermal therapy. Adv Matter.

[21] Hoter A, Naim HYJC (2019). Heat shock proteins and ovarian cancer: important roles and therapeutic opportunities. Cancer.

[22] Tang X, Tan L, Shi K, Peng J, Xiao Y, Li W, Chen L, Yang Q, Qian ZJ. Gold nanorods together with HSP inhibitor-VER-155008 micelles for colon cancer mild-temperature photothermal therapy. Acta Pharm Sin B. 2018; 8:587–601. 10.1016/j.apsb.2018.05.011 PMC6089863 30109183

[23] Mathieu C, Messaoudi S, Fattal E, Vergnaud-Gauduchon JJCDR: Cancer drug resistance: rationale for drug delivery systems and targeted inhibition of HSP90 family proteins. 2019. 10.20517/cdr.2019.26 PMC8992530 35582577

[24] Hu J-J, Liu M-D, Gao F, Chen Y, Peng S-Y, Li Z-H, Cheng H, Zhang X-Z (2019). Photo-controlled liquid metal nanoparticle-enzyme for starvation/photothermal therapy of tumor by win-win cooperation. Biomaterials.

[25] Zhou J, Li M, Hou Y, Luo Z, Chen Q, Cao H, Huo R, Xue C, Sutrisno L, Hao LJ (2018). Engineering of a nanosized biocatalyst for combined tumor starvation and low-temperature photothermal therapy. ACS Nano.

[26] Mellatyar H, Talaei S, Pilehvar-Soltanahmadi Y, Barzegar A, Akbarzadeh A, Shahabi A, Barekati-Mowahed M, Zarghami NJB. Targeted cancer therapy through 17-DMAG as an Hsp90 inhibitor: overview and current state of the art. Biomed Pharmacother. 2018, 102:608–17. 10.1016/j.biopha.2018.03.102 29602128

[27] Song Y, Wang Y, Zhu Y, Cheng Y, Wang Y, Wang S, Tan F, Lian F, Li NJ. Biomodal tumor‐targeted and redox‐responsive Bi2Se3 hollow nanocubes for MSOT/CT imaging guided synergistic low‐temperature photothermal radiotherapy. Adv Healthc Mater. 2019, 8:1900250. 10.1002/adhm.201900250 31290616

[28] Jin R, Xie J, Yang X, Tian Y, Yuan P, Bai Y, Liu S, Cai B, Chen XJBS (2020). A tumor-targeted nanoplatform with stimuli-responsive cascaded activities for multiple model tumor therapy. Biomater Sci.

[29] Chen B-Q, Kankala RK, Zhang Y, Xiang S-T, Tang H-X, Wang Q, Yang D-Y, Wang S-B, Zhang YS, Liu GJCEJ. Gambogic acid augments black phosphorus quantum dots (BPQDs)-based synergistic chemo-photothermal therapy through downregulating heat shock protein expression. Adv Mater. 2020; 390:124312.

[30] Li C, Wang X, Song H, Deng S, Li W, Li J, Sun JJ (2020). Current multifunctional albumin-based nanoplatforms for cancer multi-mode therapy. Asian J Pharm Sci.

[31] Elzoghby AO, Samy WM, Elgindy NA (2012). Albumin-based nanoparticles as potential controlled release drug delivery systems. J Control Release.

[32] Huang D, Chen Y-S, Green CR, Rupenthal IDJB (2018). Hyaluronic acid coated albumin nanoparticles for targeted peptide delivery in the treatment of retinal ischaemia. Biomaterials.

[33] Lam P-L, Kok S-L, Gambari R, Kok T-W, Leung H-Y, Choi K-L, Wong C-S, Hau D-P, Wong W-Y, Lam KJGC (2015). Evaluation of berberine/bovine serum albumin nanoparticles for liver fibrosis therapy. Biomater Sci.

[34] Liu L, Hu F, Wang H, Wu X, Eltahan AS, Stanford S, Bottini N, Xiao H, Bottini M, Guo WJAN (2019). Secreted protein acidic and rich in cysteine mediated biomimetic delivery of methotrexate by albumin-based nanomedicines for rheumatoid arthritis therapy. ACS Nano.

[35] Lin T, Zhao P, Jiang Y, Tang Y, Jin H, Pan Z, He H, Yang VC, Huang YJ. Blood–brain-barrier-penetrating albumin nanoparticles for biomimetic drug delivery via albumin-binding protein pathways for antiglioma therapy. 2016; 10:9999–10012. 10.1021/acsnano.6b04268 27934069

[36] Qu N, Lee RJ, Sun Y, Cai G, Wang J, Wang M, Lu J, Meng Q, Teng L, Wang DJ (2016). Cabazitaxel-loaded human serum albumin nanoparticles as a therapeutic agent against prostate cancer. Int J Nanomed.

[37] Liu L, Bi Y, Zhou M, Chen X, He X, Zhang Y, Sun T, Ruan C, Chen Q, Wang HJ (2017). Biomimetic human serum albumin nanoparticle for efficiently targeting therapy to metastatic breast cancers. ACS Appl Mater Interfaces.

[38] Sun X, Sun J, Lv J, Dong B, Liu M, Liu J, Sun L, Zhang G, Zhang L, Huang GJ. Ce6-C6-TPZ co-loaded albumin nanoparticles for synergistic combined PDT-chemotherapy of cancer. J Mater Chem B. 2019; 7:5797–807. 10.1039/c9tb01346f 31483422

[39] Ren H, Liu J, Li Y, Wang H, Ge S, Yuan A, Hu Y, Wu JJAB (2017). Oxygen self-enriched nanoparticles functionalized with erythrocyte membranes for long circulation and enhanced phototherapy. Acta Biomater.

[40] Hu D, Xu H, Xiao B, Li D, Zhou Z, Liu X, Tang J, Shen YJ. Albumin-stabilized metal–organic nanoparticles for effective delivery of metal complex anticancer drugs. ACS Appl Mater Interfaces. 2018; 10:34974–82. 10.1021/acsami.8b12812 30238746

[41] Chen Q, Feng L, Liu J, Zhu W, Dong Z, Wu Y, Liu ZJ. Intelligent albumin–MnO_2_ nanoparticles as pH‐/H2O2‐responsive dissociable nanocarriers to modulate tumor hypoxia for effective combination therapy. Adv Mater. 2016; 28:7129–36. 10.1002/adma.201601902 27283434

[42] Tian L, Chen Q, Yi X, Chen J, Liang C, Chao Y, Yang K, Liu ZJS (2017). Albumin-templated manganese dioxide nanoparticles for enhanced radioisotope therapy. Small.

[43] Li B, Wang Q, Zou R, Liu X, Xu K, Li W, Hu JJ. Cu 7.2 S 4 nanocrystals: a novel photothermal agent with a 56.7% photothermal conversion efficiency for photothermal therapy of cancer cells. 2014; 6:3274–3282. 10.1039/c3nr06242b 24509646

[44] Zhang W, Li S, Liu X, Yang C, Hu N, Dou L, Zhao B, Zhang Q, Suo Y, Wang JJ. Oxygen‐generating MnO_2_ nanodots‐anchored versatile nanoplatform for combined chemo‐photodynamic therapy in hypoxic cancer. Acta Biomater. 2018, 28:1706375.

[45] Yan F, Duan W, Li Y, Wu H, Zhou Y, Pan M, Liu H, Liu X, Zheng HJT. NIR-laser-controlled drug release from DOX/IR-780-loaded temperature-sensitive-liposomes for chemo-photothermal synergistic tumor therapy. Theranostics. 2016; 6:2337. 10.7150/thno.14937 PMC5118599 27877239

[46] Tian H, Zhang J, Zhang H, Jiang Y, Song A, Luan YJ. Low side-effect and heat-shock protein-inhibited chemo-phototherapy nanoplatform via co-assembling strategy of biotin-tailored IR780 and quercetin. Acta Biomater. 2020; 382:123043.

[47] Li W, Yong J, Xu Y, Wang Y, Zhang Y, Ren H, Li XJC, Biointerfaces SB (2019). Glutathione depletion and dual-model oxygen balance disruption for photodynamic therapy enhancement. Colloids Surf B Biointerfaces.

[48] Wang P, Jiang F, Chen B, Tang H, Zeng X, Cai D, Zhu M, Long R, Yang D, Kankala RKJC, Biointerfaces SB (2020). Bioinspired red blood cell membrane-encapsulated biomimetic nanoconstructs for synergistic and efficacious chemo-photothermal therapy. Colloids Surf B Biointerfaces.

[49] Ng CW, Li J, Pu KJ (2018). Recent progresses in phototherapy-synergized cancer immunotherapy. Immunotherapy.

[50] Zhang Y, Wan Y, Chen Y, Blum NT, Lin J, Huang PJ. Ultrasound-enhanced chemo-photodynamic combination therapy by using albumin “Nanoglue”-based nanotheranostics. J Mater Chem B. 2020; 14:5560–9. 10.1021/acsnano.9b09827 32343559

[51] Lim Z-ZJ, Li J-EJ, Ng C-T, Yung L-YL, Bay B-HJ. Gold nanoparticles in cancer therapy. Mol Pharm. 2011; 32:983–90. 10.1038/aps.2011.82 PMC4002534 21743485

[52] Agarwal R, Jurney P, Raythatha M, Singh V, Sreenivasan SV, Shi L, Roy KJ. Effect of shape, size, and aspect ratio on nanoparticle penetration and distribution inside solid tissues using 3D spheroid models. Adv Healthc Mater. 2015; 4:2269–80. 10.1002/adhm.201500441 PMC9346573 26376024

[53] Sharifi M, Attar F, Saboury AA, Akhtari K, Hooshmand N, Hasan A, El-Sayed MA, Falahati MJ (2019). Plasmonic gold nanoparticles: Optical manipulation, imaging, drug delivery and therapy. J Control Release.

[54] Ji X, Kong N, Wang J, Li W, Xiao Y, Gan ST, Zhang Y, Li Y, Song X, Xiong QJ. A novel top‐down synthesis of ultrathin 2D boron nanosheets for multimodal imaging‐guided cancer therapy. Adv Mater. 2018; 30:1803031. 10.1002/adma.201803031 PMC6338531 30019786

[55] Tao W, Ji X, Zhu X, Li L, Wang J, Zhang Y, Saw PE, Li W, Kong N, Islam MA (2018). Two-dimensional antimonene-based photonic nanomedicine for cancer theranostics. Adv Mater.

[56] Qiu M, Singh A, Wang D, Qu J, Swihart M, Zhang H, Prasad PN (2019). Biocompatible and biodegradable inorganic nanostructures for nanomedicine: silicon and black phosphorus. J Mater Chem B.

[57] Chen S, Xing C, Huang D, Zhou C, Ding B, Guo Z, Peng Z, Wang D, Zhu X, Liu SJ. Eradication of tumor growth by delivering novel photothermal selenium-coated tellurium nanoheterojunctions. Sci Adv. 2020; 6:eaay6825. 10.1126/sciadv.aay6825 PMC7141822 32284997

[58] Luo M, Fan T, Zhou Y, Zhang H, Mei LJ. 2D black phosphorus–based biomedical applications. Acta Biomater. 2019; 29:1808306.

[59] Xu Y, Ren H, Liu J, Wang Y, Meng Z, He Z, Miao W, Chen G, Li XJN (2019). A switchable NO-releasing nanomedicine for enhanced cancer therapy and inhibition of metastasis. Nanoscale.

[60] Chen Q, Liu ZJAM (2016). Albumin carriers for cancer theranostics: a conventional platform with new promise. Adv Mater.

