# Supplementary material for: Synergy of hypoxia relief and heat shock protein inhibition for phototherapy enhancement
Source: J Nanobiotechnology. 2021 Jan 6;19:9. doi: 10.1186/s12951-020-00749-5 (PMC7789325; doi:10.1186/s12951-020-00749-5)
Supplement: Supplementary file 1 — Additional file 1: FigS1-FigS3. The main characteristics (zeta potential, XPS and FT-IR) of IGM nanoparticles. Fig. S4. Standard line of GA determined by UV-vis spectrophotometer. Figs. S5–S6. The size distribution of IGM and stability of IR780 in different temperature. Fig. S7. The mean fluorescence intensity of intracellular hypoxia. Fig. S8. The cell viability of HUVEC and 4T1 cells after incubation with IGM. Fig. S9. The immunofluorescence staining and mean fluorescence intensity of Hsp90 in 4T1 cells after various treatments. Fig. S10. IR thermal images of tumor bearing mice with NIR laser irradiation. Fig. S11. The mean fluorescence of hypoxia in tumor after intravenous injection of saline and IGM. Fig. S12. The mean fluorescence of Hsp90 in tumor after various treatments. [file 12951_2020_749_MOESM1_ESM.docx]

**Additional information**

**Synergy of Hypoxia Relief and Heat Shock Protein Inhibition for Phototherapy Enhancement**

Gutian Zhang^1^*, Wenting Cheng^2^, Lin Du^3^, Chuanjun Xu^2^, Jinlong Li^2^*

1. Department of Urology, Drum Tower Hospital, Medical School of Nanjing University, Nanjing 210008, China

2. Department of Laboratory Medicine, the Second Hospital of Nanjing, Nanjing University of Chinese Medicine, Nanjing 210003, China.

3. Department of Urology, Drum Tower Hospital Medical School of Southeast University, Nanjing 210008, China

*Author for correspondence:

Dr. Jinlong Li, PhD.

E-mail: [lijinlong1028@126.com](mailto:lijinlong1028@126.com)

Dr. Gutian Zhang, PhD.

E-mail: zhang.gutian@nju.edu.cn


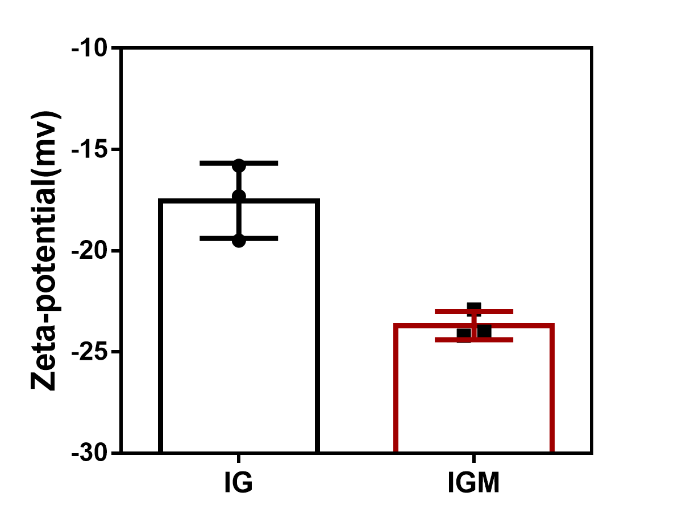


**Figure S1.** The zeta potential of IG and IGM.


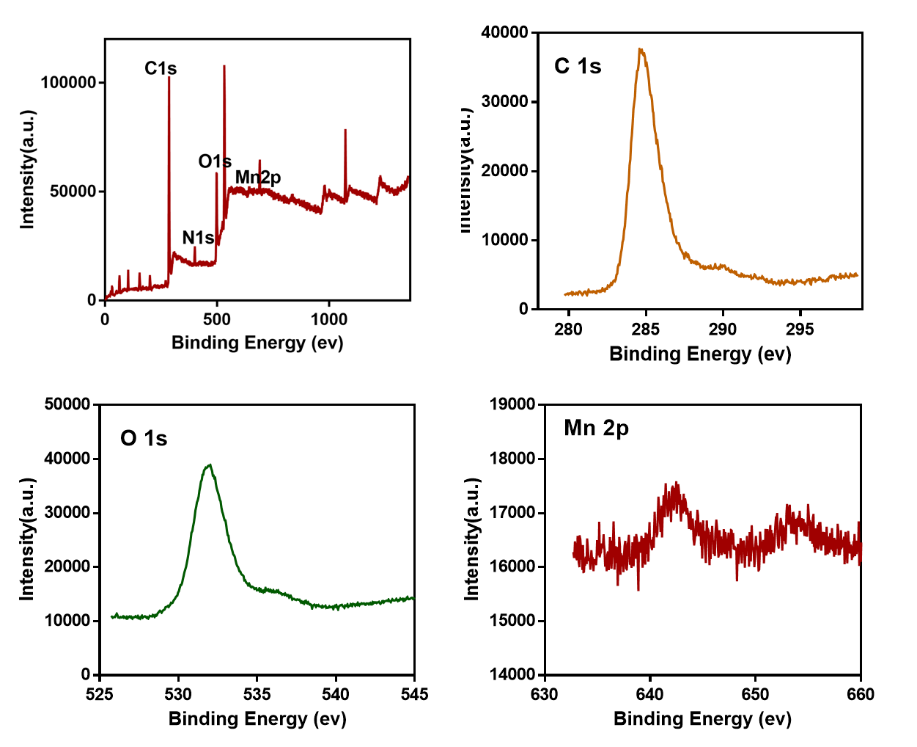


**Figure S2**. X-ray photoelectron spectroscopy survey and spectrum of main elements in IGM.


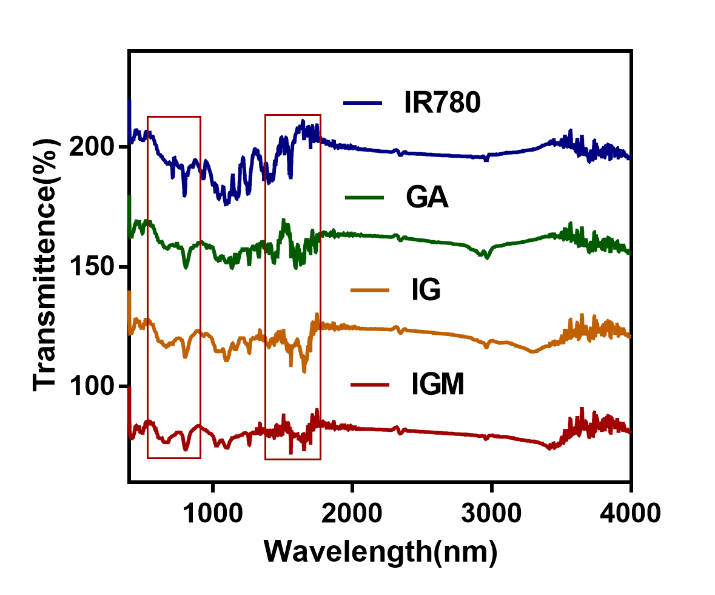


**Figure S3**. FT-IR spectra of IR780, GA, IG and IGM


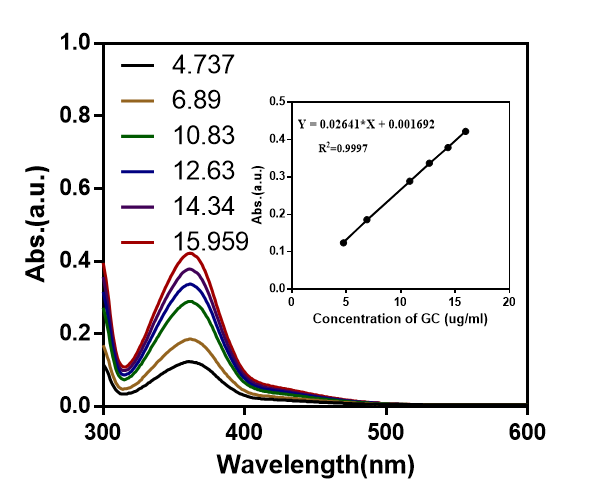


**Figure S4**. Standard line of GA determined by UV-vis spectrophotometer


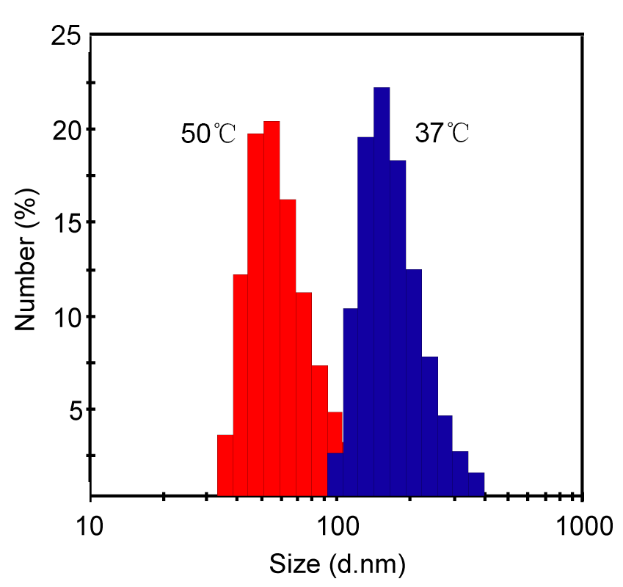


**Figure S5.** The size distribution of IGM in different temperature (37 and 50 ℃)


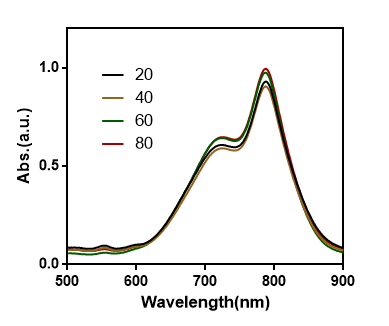


**Figure S6.** The photostability of IR780 under different temperature (20, 40, 60 and 80℃) for 5mins.


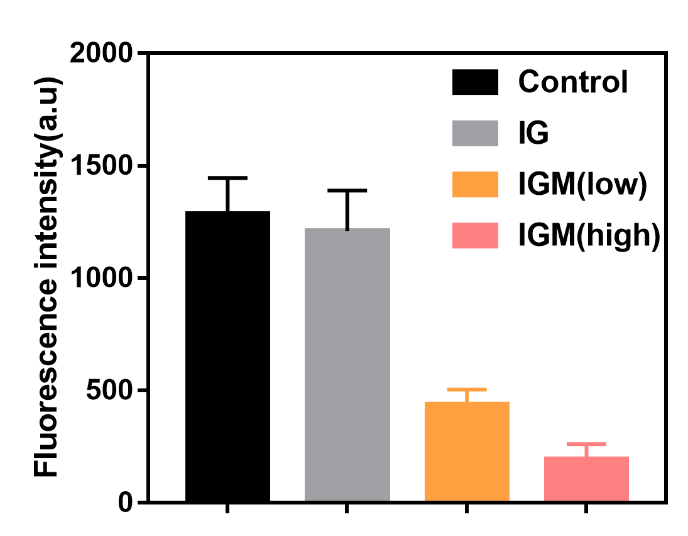


**Figure S7.** The mean fluorescence intensity of intracellular hypoxia


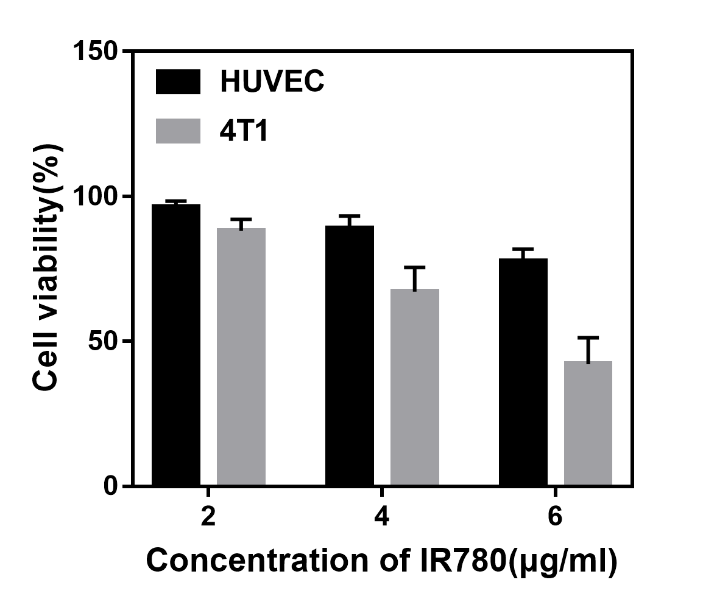


**Figure S8.** The cell viability of HUVEC and 4T1 cells after incubation with IGM.


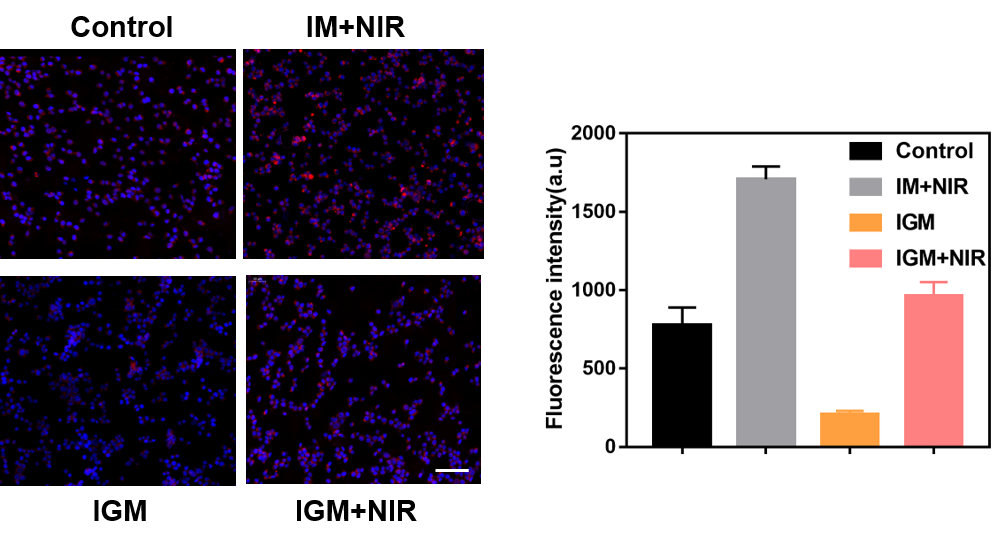


**Figure S9.** The immunofluorescence staining and mean fluorescence intensity of Hsp90 in 4T1 cells after various treatments


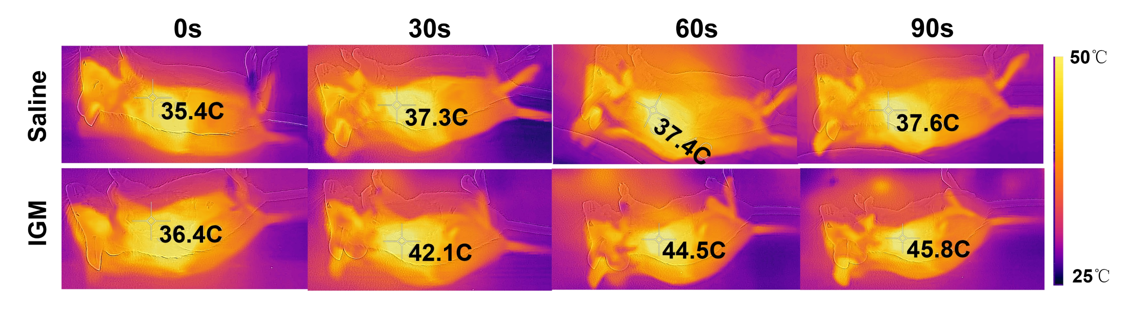


**Figure S10.** IR thermal images of tumor bearing mice with NIR laser irradiation


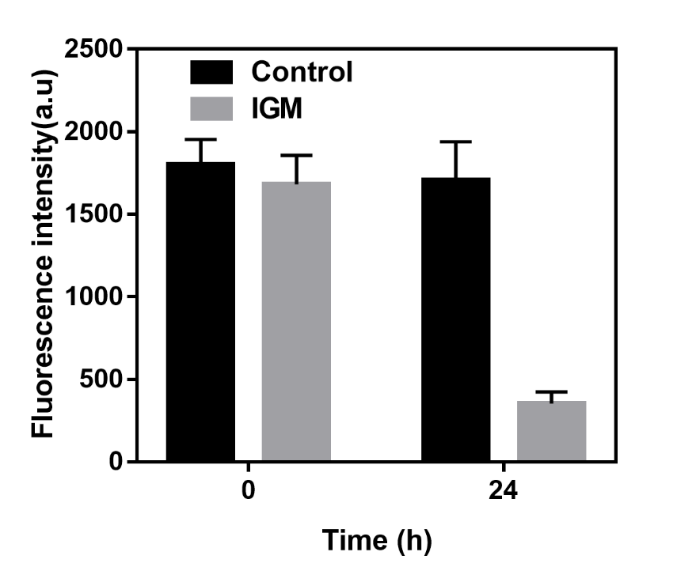


**Figure S11**. The mean fluorescence of hypoxia in tumor after intravenous injection of saline and IGM


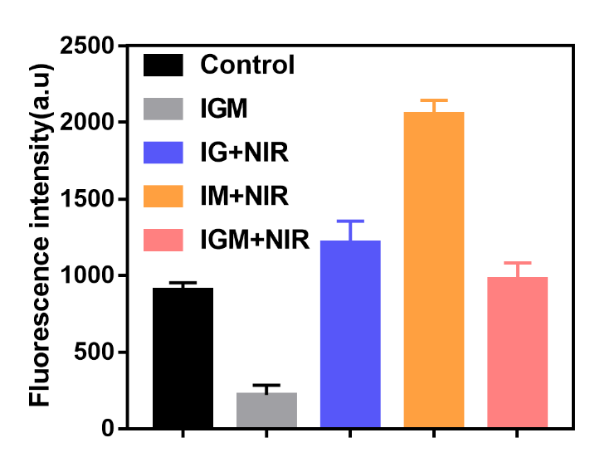


**Figure S12.** The mean fluorescence of Hsp90 in tumor after various treatments.
